# An Aquatic Mobile Sensing USV Swarm with a Link Quality-Based Delay Tolerant Network

**DOI:** 10.3390/s18103440

**Published:** 2018-10-13

**Authors:** Daniela Sousa, Miguel Luís, Susana Sargento, Artur Pereira

**Affiliations:** 1 Instituto de Telecomunicações, 3810-193 Aveiro, Portugal; nmal@av.it.pt (M.L.); susana@ua.pt (S.S.); 2 Departamento de Eletrónica, Telecomunicações e Informática, University of Aveiro, 3810-193 Aveiro, Portugal; artur@ua.pt

**Keywords:** aquatic mobile sensing platform, unmanned surface vessels, delay tolerant network, forwarding strategies, link quality estimator, simulation and real experimentation, low-cost systems

## Abstract

The Smart City concept is starting to extend into maritime environments alongside with the increase of Unmanned Surface Vehicles (USV) models on the market. Consequently, by joining both Smart City and USV technologies, a set of platforms and applications for aquatic environments are emerging. This work proposes a low-cost aquatic mobile sensing platform for data gathering with a swarm of USVs communicating through a Delay-Tolerant Network (DTN). A set of DTN link quality-based routing strategies select the best quality path in a dynamic approach so the sensed information is able to reach the mobile gateway in a reliable way. A Link Quality Estimation (LQE) approach is proposed and its accuracy is evaluated through real experimentation. An aquatic simulation environment, considering both navigation and communication layers, was also proposed and used to evaluate the performance of the proposed routing strategies, and complement real environment performance studies.

## 1. Introduction

Over the past decade, to reduce human effort and increase efficiency, Unmanned Surface Vehicles (USVs) and other low-cost systems have been deployed in both military and civilian applications. Aquaculture is one of the civilian applications that mostly uses this type of solutions for aquatic monitoring, playing an important role in the monitoring and detection of dangerous levels of water quality. However, available USV platforms have low payload capacity and short endurance times. To overcome these issues, the deployment of a cooperative formation fleet, also known as swarm of USVs equipped with sensors, can be used, improving robustness and increasing fault-tolerant resilience [1]. In addition, a group of cooperative USVs (mobile robots) can achieve a better overall performance in tasks such as exploration, coverage and cooperative sensing [2].

These solutions call for a dynamic and decentralized architecture for location decisions between the USVs, and communications play a vital role for their interaction and gathering of the sensors’ data. These communications require low latency, bandwidth efficiency, and high adaptability in the network, to cope with the mobility of the USVs and the unstable network. With an increasing number of USVs in range, a multi-hop network emerges, capable of high traffic communications, where no centralized authority is necessary and where each node can enter or leave the network, thus ceasing to exist communication with the destination on some occasions. To overcome the isolated node limitation in the network and cope with the lack of continuous network connectivity, a Delay-Tolerant Network (DTN)-based approach can be used.

The mobility of the network topology may decrease the time for communication and gathering the data; therefore, it is important to maximize the quality of the communication. This can be achieved by finding and using the links and the communication routes with the best quality, thus improving the performance in terms of latency, delivery ratio, and bandwidth, among others. For the success of this type of routing, accurate link quality estimation is essential. Although several Link Quality Estimators (LQE) have been proposed over the past years [3], many approaches rely on active probing, causing more overhead in the network, or overhearing all the packets (even if the node is not the destination) to infer the Received Signal Strength (RSSI), which is also expensive.

In this paper, we propose a platform for data gathering in aquatic monitoring with a swarm of USVs communicating through DTN routing using a passive link quality estimation approach, i.e., a link quality estimation based on a monitoring process that does not disturb the network (in terms of collision and energy consumption). Moreover, since in aquaculture the water tanks can be off the coast with no Wi-Fi or other short range communication, this platform integrates multiple technologies (both long and short range communications) for a better real-time data gathering. The proposed approach is both tested in a real environment to assess the real link quality of the network, and in a simulated environment to assess the performance of the approach with a swarm of USVs.

The main contributions of this work can be summarized as follows:

1. Aquatic monitoring platform through a swarm of USVs;
2. Data collection units with aquatic environment monitoring;
3. Passive link quality estimation;
4. DTN routing strategies through the best quality path in a dynamic approach;
5. DTN supporting a mobile sensing network and multi-technology communication (both long and short range communications); and
6. Network and path simulation in Robot Simulator (ROS) which can be integrated with real USVs in a real environment.

The remainder of this article is organized as follows. Section 2 discusses the related work and positions our own work. Section 3 describes the platform architecture, the network elements, and the network functionalities, while Section 4 presents the passive link quality estimation DTN routing strategies. Section 5 presents the results of link quality estimation in real environments, and Section 6 discusses the performance results in the simulated swarm of USVs. Finally, Section 7 enumerates the conclusions and future work.

## 2. Related Work

This section describes related work in the research areas that are relevant for the proposed approach.

### 2.1. USV Platforms

Several previous works have developed USVs for aquatic monitoring and marine environments. Guobao et al. [4] stated that most efforts are related to general ocean sensing and water quality monitoring, while this work focuses on fish farming where there are not many technological solutions. Lloret et al. [5] proposed a group-based underwater wireless sensor network to monitor the fecal waste and uneaten feed deposited on the seabed. The nodes’ mobility, grouped with the number of nodes, give them a coverage of 100% of the tanks. López et al. [6] presented a centralized approach based on ZigBee with a master–slave architecture to measure temperature and Potential of Hydrogen (pH) levels. They also developed a power consumption algorithm to improve the node lifetime. Bhadauria et al. [7] developed a robotic raft with searching and fish tracking algorithms which, in several aquatic environments, has shown to have good results. Saravanan et al. [8] presented an IoT-based system to process the tracking, collect, and monitor the water quality. Each node is equipped with sensors, a microcontroller and Long Range (LoRa) capabilities. The system has an alert triggering mechanism to alarm the authorities in the case of any changes in water quality, similar to the work in [9], although not applied to USV systems.

None of the previous approaches considered the interaction between the USVs in the swarm to optimize the aquatic monitoring process. Our proposal is to develop a robust swarm of robots with monitoring capabilities in an IoT platform with multiple communication technologies, with opportunistic and delay tolerant communications, giving space for a multi-hop data forwarding approach.

### 2.2. Link Quality Estimators

Link Quality Estimators (LQEs) such as LQI (Link Quality Indicator), RSSI (Received Signal Strength Indicator), and SNR (Signal-to-Noise Ratio) are considered hardware based, because they are directly read from the transceiver, not requiring any additional computation. However, as shown in previous studies, hardware based estimators are inaccurate [10]. Software based estimators enable the count of the reception ratio or the average number of packet transmissions/re-transmission. Some of the estimators explained in [10] are presented next.

PRR (Packet Reception Ratio) counts the average number of packet retransmissions required before a successful reception. It is recommended for applications requiring low complexity level with moderate performance. Expected Transmission Count (ETX) tries to estimate the number of transmissions that are necessary to send a packet successfully. The number of packets received within a fixed window is compared to the number of packets expected, only updating the value at the end of each window. Four-Bit approximates the average number of packet transmissions/re-transmissions before a successful reception, but it heavily depends on the tuning of its parameters.

Fuzzy Link-Quality Estimator (F-LQE), proposed by Baccour et al. [11], uses SNR, PSR (Packet Success Ratio), link asymmetry level (ASL), and stability (SF). The evaluation of the fuzzy rule returns the membership of the link in the fuzzy subset of good links. However, fuzzy methods incur a loss of precision despite being faster. Holistic Packet Statistics (HoPS), proposed by Renner et al. [12], calculates four distinct quality metrics, describing the short- and long-term quality of a link at the same time, while also providing information about the dynamics of a link by means of the variation and trend of the link-quality. However, it is only possible to predict a useful link quality if the link behavior is studied in more detail. Wavelet-Neural-Network-based Link Quality Estimation (WNN-LQE) was presented by Sun et al. [13] and uses SNR and PRR, but has a complex process for the estimation, not making it easier to implement in WSN nodes.

In wireless networks, a routing protocol should select the shortest path with the most reliable paths [14], minimizing the link layer transmissions for a packet to be delivered and acknowledged. Dawans et al. [15] demonstrated that, using a default value for new neighbors tends to favor these neighbors among other connections; thus, the link quality of a connection has to be continuously estimated.

Our network is used for navigation, environmental data gathering, and dissemination purposes, being considered a dense network. Therefore, an active monitoring approach may not be feasible, due to load and latency increase. Due to hardware characteristics, the overhearing technique is expensive, because the node must capture all the packets in the network in order to retrieve the RSSI of the received frame, and also time consuming since the wireless interface must be in monitoring mode. Therefore, our proposal is to use a passive monitoring for link quality estimator, without increasing collisions, network overhead and energy consumption.

### 2.3. DTN Forwarding Strategies

Traditional routing protocols demand a stable end-to-end connection between source and destination, failing in challenging environments. Delay tolerant networks experience high heterogeneity and volatility in the network, not always having end-to-end connection. Routing protocols for this type of architecture have to adapt to challenging environments. The developed routing protocols over the years present a trade-off between controlled replication and network knowledge. On the one hand, a pure replication protocol, such as flooding, uses high resources due to the broadcasting of the packets to all vicinity nodes, leading to high network congestion. On the other hand, a pure knowledge protocol requires high resources to process and gather the information, while maintaining an updated routing table [16].

The Epidemic protocol implements a replication protocol flooding the bundle through the network. It does not require any prior knowledge [17]. This protocol increases the network overhead, since the packet can reach the destination by multiple paths. The Direct Contact protocol only delivers the packet when the source is directly connected to the destination; it has a small network overhead, but a high delay, and a low delivery ratio [18].

Moura et al. [19] proposed a DTN-based routing through LQE that uses a hybrid monitoring, through active probing and overhearing. This LQE uses RSSI, link stability and available bandwidth. Almeida et al. [20] proposed Q-PRoPHET, a quality-based routing protocol capable of performing multi-hop routing decisions based on the paths’ qualities. This protocol is based on PRoPHET and uses a decay function and a transitive probability, avoiding sending replicas to poor link qualities. The LQE used is based on RSSI and link stability, making it an active monitoring estimator.

QoN-BSW presented by Wang et al. [21] is an improvement of the Binary Spray and Wait protocol, since it improves the delivery rate, reduces the average delay, and reduces the network overhead. This protocol presents the notion of QoN, that is, the number of nodes met in a period of time. In the first phase, when a node encounters another, they will update their QoNs and then exchange QoNs with each other. After this, the amount of message copies that are forwarded takes into account the QoN number of the destination node. In the wait phase, if a node only has one copy left, it switches to direct transmission and only forwards this message to the destination. The Hybrid of Probability and message Redundancy (HPR) [22] routing algorithm is based on a combination of packet delivery ratio (PDR) and message redundancy, with the aim of reducing the communication overhead while keeping the high message delivery ratio. This algorithm estimates the delivery probability of the node based on the history of encounter information and contact duration to provide a more precise and reasonable estimation of delivery probability.

Our proposal is to develop a software-based LQE with only passive monitoring, combining multiple metrics. To have a better understanding of each protocol, and also our proposed LQE Passive Multihop Link Quality Estimator (PAmuLQE), Table 1 summarizes the differences and similarities between them.

### 2.4. Simulation

There are several simulators for networking, and for robotics. Pessoa et al. [23] presented mOVERS, an emulator capable of recreating scalable vehicular scenarios of data gathering and content distribution in vehicular networks. This emulator works with mobile Opportunistic VEhicular (mOVE), a DTN-based architecture supporting communications using IEEE 802.11p and Wireless Access in Vehicular Environments (WAVE), and IEEE 802.11a/b/g (Wi-Fi technology) developed in the Network Architectures and Protocols research group www.it.pt/Groups/Index/36, where each node is capable of storing a packet and forwards it when a neighbor is available. Keränen et al. [24] presented the Opportunistic Networking Environment (ONE) simulator specifically designed for evaluating DTN routing and application protocols. It uses movement models to create scenarios, and already includes six well-known routing protocols. Since these platforms do not support robotic and physical simulation, they do not allow testing path planning or control algorithms.

Simulators for aquatic robotics are categorized in two areas: underwater and surface. The majority of the simulators developed lies on the underwater category, such as the open source SubSim [25], Neptune [26], IGW [27] and MVS [28].

**Figure 1:**
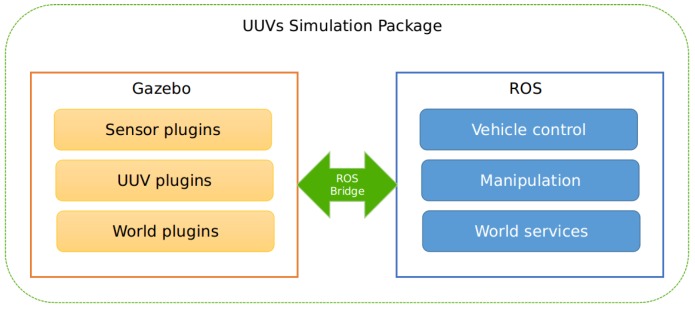
UUV Simulator: Software structure inspired by Manhães et al. [31].

Mendonça et al. [29] described a ROS and Gazebo based simulator, named *Kelpie*, for testing and debugging real hardware of aquatic robots, providing more accurate physics simulation and rendering quality by using the geometric meshes of the vehicle models instead of simple geometric shape. Kelpie also allows the simulation of Unmanned Aerial Vehicles (UAVs). Santos et al. [30] described a framework for the simulation of multiple water surface vehicles operating simultaneously. This testing tool requires modules to be developed in C++. Manhães et al. [31] presented the *UUV Simulator*, which is an open source gazebo-based project, with several algorithms for Unnamed Underwater Vehicles (UUVs). This project implements design models, world models, sensor models, and control algorithms. It is a simulator that allows hydrodynamic constraints, multi-robot systems, and the integration of new modules. This package also integrates an extensive wikipedia on all the modules and tutorials to integrate new robots, worlds or other control algorithms. Because it is ROS and Gazebo based, it is easily replicated in a RaspberryPi with sensors, facilitating the translation from simulated code to the real scenario. Due to these aspects, this package was chosen for the development of a new ROS and Gazebo based simulator with a network module for Unnamed Surface Vehicles. An overview of the modules included in this package is depicted in Figure 1.

## 3. Proposed Architecture

This Section presents the proposed architecture, along with the software modules and hardware used to create an aquatic monitoring platform, capable of collecting environmental and quality data from the sensors and send it to a remote server. Section 3.1 overviews the proposed network architecture along with its requirements, and Section 3.2 presents the network elements along with its hardware and software characterization.

### 3.1. Architecture Overview

**Figure 2:**
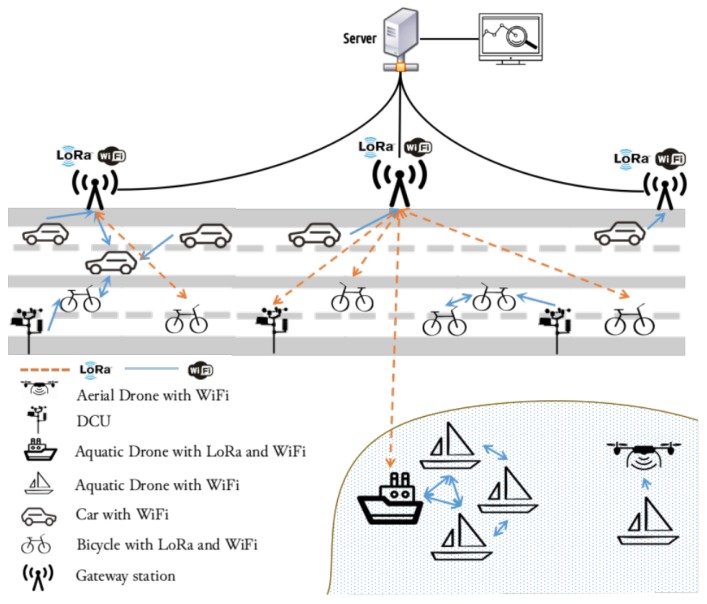
City-wide platform overview [16].

The aquatic monitoring platform is a part of a larger city-wide architecture described by Almeida et al. [16] and illustrated in Figure 2. This platform contains heterogeneous elements, such as Data Collecting Units (DCUs), cars, bicycles, aerial drones and USVs. The aquatic platform is integrated in the more general one, but has specific requirements which require an extended architecture:

- Heterogeneous mobile nodes (USVs), with only short range communication (e.g., Wi-Fi) or also long range communication (e.g., LoRa);
- Collection and dissemination of environmental data from the short range communication mobile nodes to the long range ones (mobile gateways);
- Fallback support of mobile aerial gateways if USVs are isolated; and
- Monitoring of an entire tank with minimal cost.

### 3.2. Network Elements

Each mobile node and gateways are composed by a Raspberry Pi board as the central processing unit with the specifications described in Table 2.

USVs have a 64-bit Ubuntu Mate + ROS operating System, while the rest of the nodes carry a 64-bit Raspbian GNU OS [16]. LoRa communication is acquired with the SX12772 LoRa module and Multiprotocol Radio Shield manufactured by Libelium [32]. The hardware chosen provides a low-cost hardware base and a modular structure, easy to repair.

The Aquatic Swarm is comprised of heterogeneous USVs (with different sensors, and communications technologies) to lower the cost and the energy consumption on each USV. Each element is fundamental to the monitoring task, since each has a different set of sensors, but they are also fundamental to the data forwarding process in the delay-tolerant network, where intermediate nodes act as relays, storing and forwarding the packets in a situation of lack of end-to-end connectivity.

The multi-technology capabilities allow the swarm to maintain communications with at least one mobile gateway with long range communication, and therefore provide the connection to the server for a longer period of time. LoRa plays an important role in the platform, since it is the only communication interface that can reach the server in the infrastructure, maintaining the real time monitoring task. LoRa has a 1% imposed regulatory duty-cycle (i.e., 36 s per hour), so, to maximize the time that the swarm can send information over LoRa, only one USV, the mobile gateway, can be transmitting data at each time, creating a bigger time window to transmit. When this communication is also not possible or a mobile node is isolated, aerial drones can be sent to gather the data.

USVs are composed of a heterogeneous set of sensors with the purpose of collecting aquatic environmental information. In total, the various sets can collect the following environmental variables:

- potential of Hydrogen (pH);
- Water Temperature;
- Salinity;
- Depth;
- Turbidity; and
- Electrical Conductivity.

**Figure 3:**
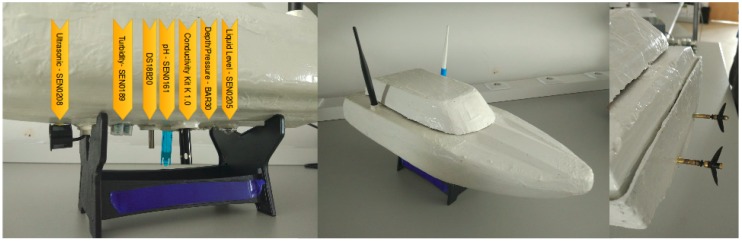
Prototype of a USV model.

**Figure 4:**
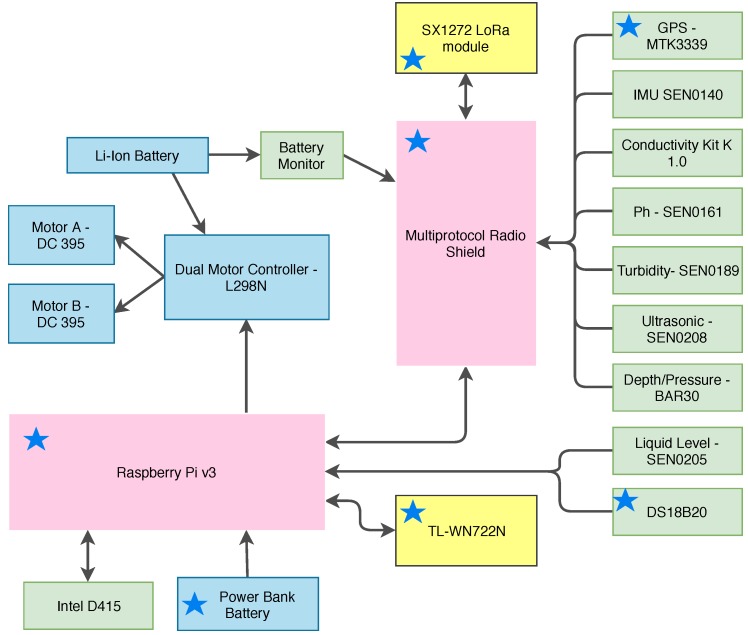
Block diagram of the USV.

An Inertial Measurement Unit (IMU), a Global Positioning System (GPS), and a camera are also added to provide information for the USV navigation system. A prototype of a USV model is presented in Figure 3 and its corresponding block diagram in Figure 4.

The model consists in a Li-Ion Battery, connected to a *Dual Motor Controller-L298N* for controlling the speed and rotation direction of two *DC 395 Boatman* Motors. This battery is also connected to a Battery Monitor in order for the user to know how much energy the robot has left for the motors. The motors are controlled by a Pulse Width Modulation (PWM) sent by the *Raspberry Pi* to the *Dual Motor Controller*. The *Raspberry Pi* and all the sensors are powered by the *Power Bank Battery*. We chose two batteries to keep the *Raspberry Pi* functional for a rescuing mission, which may be necessary if the motors can no longer run.

Some models employ an *Intel Camera D415* for mapping the area while navigating. The *Raspberry Pi* is connected to a *USB Adapter TL-WN722N* to have a higher range of Wi-Fi compared to the on-Board Wi-Fi card. The *Multiprotocol Radio Shield* is connected to the *SX1272 LoRa* module and all the sensors listed in Figure 4, and also provides a set of analog pins. Digital sensors and 1-wire sensors, e.g., *Liquid Level-SEN0205* and *DS18B20*, respectively, are directly connected to the *Raspberry Pi*.

The modules with a star in Figure 4 are the ones shared with the setup in Almeida et al. [16]. A detailed list of sensors used in the aquatic platform is presented in Table 3, however other low-cost sensors could also be used [33,34].

**Figure 5:**
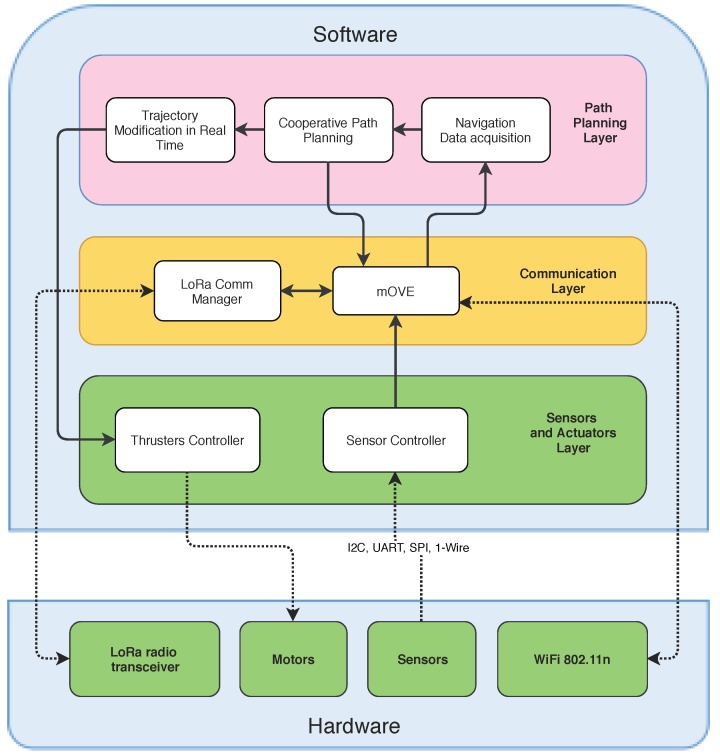
USV architecture.

The USV software architecture, described in a hierarchical structure, is presented in Figure 5. The structure consists on three major layers, i.e., Path Planning Layer, Communication Layer, and Sensors and Actuators Layer, all implemented in ROS. The Path Planning Layer plans a feasible trajectory for the USV based on the general state of the mission. The mission, generally, can be a set of interest points to be visited and sensed by the swarm. A trajectory is defined by a set of waypoints from the USV’s current position to the interest point. Since each USV is traveling in a dynamic environment, path re-planning is also needed to avoid moving obstacles, such as other USVs, and to switch destination points in order to maintain connectivity or to be more efficient at sensing all the interest points. Cooperative behavior for path planing is vital, and each USV should establish communication to ensure that it has all the swarm’s navigation information. This task is handled by the Communication Layer. This layer is responsible for exchanging packets between all nodes and also the stations in the infrastructure. Generated paths will then be passed to the Sensors and Actuators Layer to calculate specific control commands for the USV.

In more detail, a brief description of each module in the USV is described below:

- **Navigation Data Acquisition:** This module is responsible for the update and synchronization of the swarm’s navigation information given by the Communication Layer. Only the newest messages should be allowed to change position values, leading to less noise. This module is also responsible for updating the map using sensor fusion.
- **Cooperative Path Planning:** This module manages the allocation of new sensing points, and calculates the trajectory avoiding obstacles on the map. The trajectory should be optimized in terms of total distance, connectivity constraints, and navigation time. The path planning algorithm can be viewed as a multiple traveling salesmen problem, and uses a *follow me* approach, where each USV has to maintain connection with only one vehicle with a higher priority. Each USV only has to follow one vehicle and can only be followed by one vehicle. The Path Planning algorithm should maintain the connection of all the USVs in the swarm, so the swarm can be synchronized while performing a task. If one USV goes away from the swarm, another vehicle has to follow to maintain the connection between the swarm and this USV. When a USV stops communicating to the swarm, the lower priority USV drops its task and goes to the last location known of that vehicle. In a worst case scenario, all USVs’ trajectories can be affected, and the swarm starts adjusting the trajectory to follow this USV to maintain connectivity. If, for some reason, the USV has no longer connectivity with the swarm, the packets are stored until new connections are established. At that time, if the packet reaches the expiration date, then the packet is deleted. This module and the rationale behind the swarm control and navigation are still under development. In this work we have decided to focus on the evaluation of communication strategies in an aquatic sensing platform formed by moving USVs. After each allocation of a new target, this module passes this information to the communication layer to be transmitted to other USVs, using the neighbors’ announcements.
- **Trajectory Modification in Real Time:** This module is responsible for changing the trajectory to the assigned target when it is necessary to deviate from some mobile obstacle, or to maintain network connectivity. The avoidance of moving obstacles is made by integrating the on board sensors’ information and the shared positions. By fusing these data, we can reduce the noise of this information. This module is also responsible for communicating the next waypoint to the software structure shown in Figure 1 contemplated within the Thruster Controller module.
- **LoRa Comm Manager:** This module deals with LoRa technology, since the Wi-Fi is managed inside the DTN operation Processes.
- **mOVE:** This module is responsible for implementing the DTN architecture, and also managing the routing algorithm. The embedded Wi-Fi manager is responsible for finding neighbors and storing data until a neighbor is available.
- **Sensor Controller:** This module manages all the sensors’ drivers, forwards the data to the respective modules, collects the data, and fuses it to filter out noise.
- **Thrusters Controller:** This module uses the software from the *UUV Simulator* for the simulation, and also implements drivers for the real motors in the USV. It takes a waypoint and it is responsible for navigating the USV towards that point.

The software and hardware were designed in a modular architecture to give versatility and adaptability to the drone, so that the addition of new functionalities and sensors can be accomplished easily.

In this platform, beyond the mobile gateways that allow the data gathering inside the swarm to the infrastructure, there are the infrastructure gateway stations that are connected to a server that collects the data, stores it in a database for the use in other platforms, and processes the data. These stations also have the mOVE and LoRa Comm Manager modules in order to communicate with the mobile network elements and provide the DTN end-to-end path.

## 4. Link Quality-Based Forwarding Strategies

The DTN forwarding strategies here proposed aim to select the best path for a packet based on the quality of the different available links. For that, the network needs to assess the quality of each link, from every node to the mobile gateway. This section describes the estimation process of each communication link and the metrics involved.

### 4.1. Link Quality Estimation

The LQE is a core point for the proposed forwarding algorithms, since it defines the path for the transmission of each packet. This work introduces the Passive Multihop Link Quality Estimator (PAmuLQE) where each node is able to evaluate all the network links in the swarm—having the mobile gateway as destination—to find the least delay path to the gateway (being a LoRa gateway or a Wi-Fi gateway). To that end, all nodes spread their adjacency graph with the classifications through the neighbors’ announcements, making this process a passive monitoring.

This evaluation process classifies a mobile node according to the following information: (i) the estimated Signal Strength Indicator (SSI) between two nodes; and (ii) the bit rate computed with the observed delay on each received packet. With this information, a weighted factor is added in the adjacency graph, computed as follows

(1) Weight=100−Link_Quality×100,

where Link_Quality is given by

(2) $$Link\_Quality=\left(1-\beta \right)\times SSI\_est\_norm+\beta \times \frac{\sum_{i=0}^{Npackets-1}BitRate\_norm\left(i\right)\cdot Age\_factor\left(i\right)}{\sum_{i=0}^{Npackets-1}Age\_factor\left(i\right)},$$

where the SSI_est_norm is the estimated normalized value of SSI (estimation of RSSI given the distance between two USVs). By estimating the RSSI, instead of using hardware and Wi-Fi packets to measure it, we are saving resources (processing time and energy), turning this into a passive link quality estimator. BitRate_norm represents the normalized bit rate, which in turn is computed by

(3) $$BitRate=\frac{pkt\_size}{pkt\_delay}.$$

β, expressed between 0 and 1, represents a confidence level on each estimation and is given by

(4) $$\beta =\frac{\sum_{i=0}^{Npackets-1}Age\_factor\left(i\right)}{Npackets},$$

where Age_factor is given by

(5) $$Age\_factor\left(i\right)=1-\frac{min[(time\_now-time\_received(i)),MAX\_AGE]}{MAX\_AGE},$$

where time_received(i) is the time that the packet *i* was received by the node. The older a packet is, the lower the age factor is, lowering the importance of that packet to the mean. MAX_AGE is the maximum age allowed to a packet to be kept in the DTN storage.

### 4.2. Forwarding Strategies

The process of mapping the obstacles and the surrounding is accomplished in a decentralized manner to make it faster, and, in the case of failures, the swarm can maintain that functionality. In addition, all navigation decisions have to be synchronized between the USVs, which means that it is important to have a network connectivity as reliable as possible with the minimal delay between the USVs. Due to navigation issues, one of the network constraints is that all USVs are always in communication with each other.

Additionally, the aquatic mobile sensing platform should be able to get information about the water quality and send it back to a centralized unit for further processing as quickly and reliably as possible, i.e., without dropping any sensing measurement. Therefore, the routing strategy should present the lowest delay, introduce the least overhead to the network, maintaining a 100% delivery ratio, and be adaptable to the nodes’ mobility. With this in mind, a link quality-based routing strategy with three variants regarding the acknowledgment process are proposed:

- Passive multihop Link Quality Estimator with broadcast end-to-end acknowledgement (PAmuLQE-B-E2E ): In this variant, the destination node sends the acknowledgment packet in broadcast to all its neighbors. If the same packet is transmitted to a neighboring node that has the knowledge that this packet has already been delivered, this neighbor will send and acknowledge the packet in unicast to the sender node.
- Passive multihop Link Quality Estimator with unicast end-to-end acknowledgement (PAmuLQE-U-E2E): In this variant, the end-to-end acknowledgement is sent as a data packet in unicast. When the packet is retransmitted from the sender to the gateway, it is deleted from a relay node every time that the next hop transmission is acknowledged.
- Passive multihop Link Quality Estimator with neighbor acknowledgement (PAmuLQE-NACK): In this variant, each node keeps the packet only until it receives the acknowledgement from the next node. This means that there is only one copy of the packet in the network at each time.

**Figure 6:**
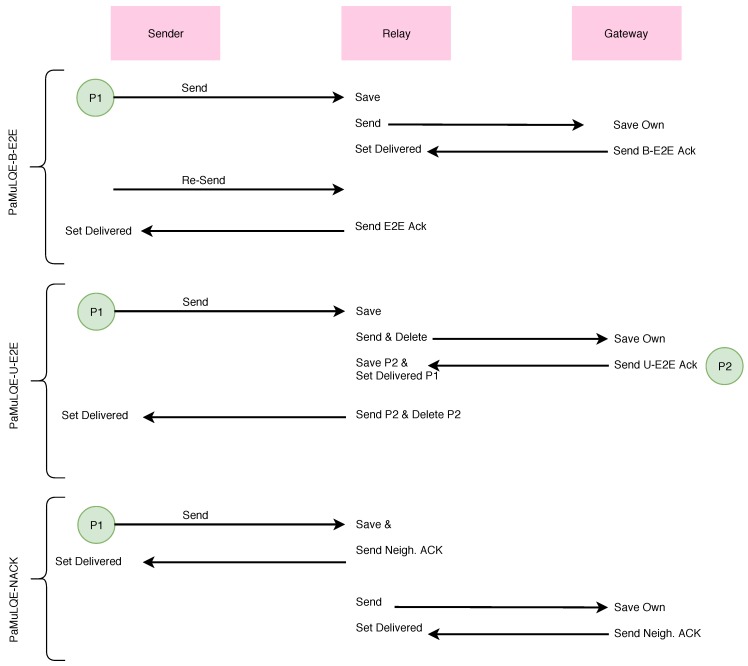
Routing logic for the three variants of the link quality-based routing strategy.

The logic of the three variants is described in Figure 6.

Algorithm 1 represents the rationale behind the link quality-based proposed forwarding decision. The routing decision strategy is common to all, as can be seen in Algorithm 1. That is, if the destination is a direct neighbor, then the packet is sent directly to the destination (Lines 4 and 5). If it is not, then it removes all neighbors that already have the bundle from the possible paths (Line 7) to employ a loop avoidance technique, by keeping tracking information, i.e., keeping the list of previous hops (previous nodes), and not sending to those hops again, controlling the amount of network resources consumed and reducing the number of copies in the network. Then, it finds the best path using the Dijkstra algorithm and the weights computed by Equation (1) (Line 8). If there is a path, then the packet is sent to the next hop given by that solution (Line 10). Line 11 introduces the differences between PAmuLQE-U-E2E and the remaining variants of the proposed strategy.
**Algorithm 1:** Routing decision algorithm (decision logic).
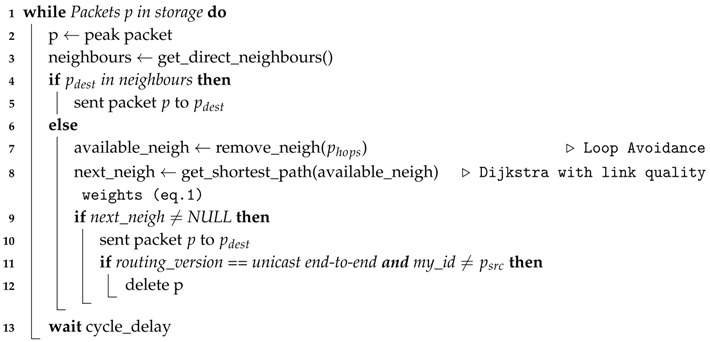


Algorithm 2 describes the logic behind the acknowledgement process for the proposed routing strategy, where Line 3 differentiates each variant of the PAmuLQE strategy.
**Algorithm 2:** Acknowledgement logic.
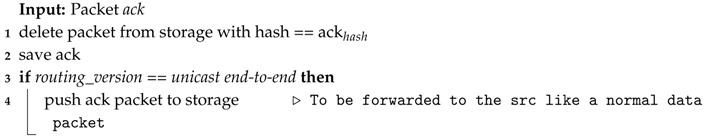


## 5. Link Quality Characterization for Simulation Support

**Figure 7:**
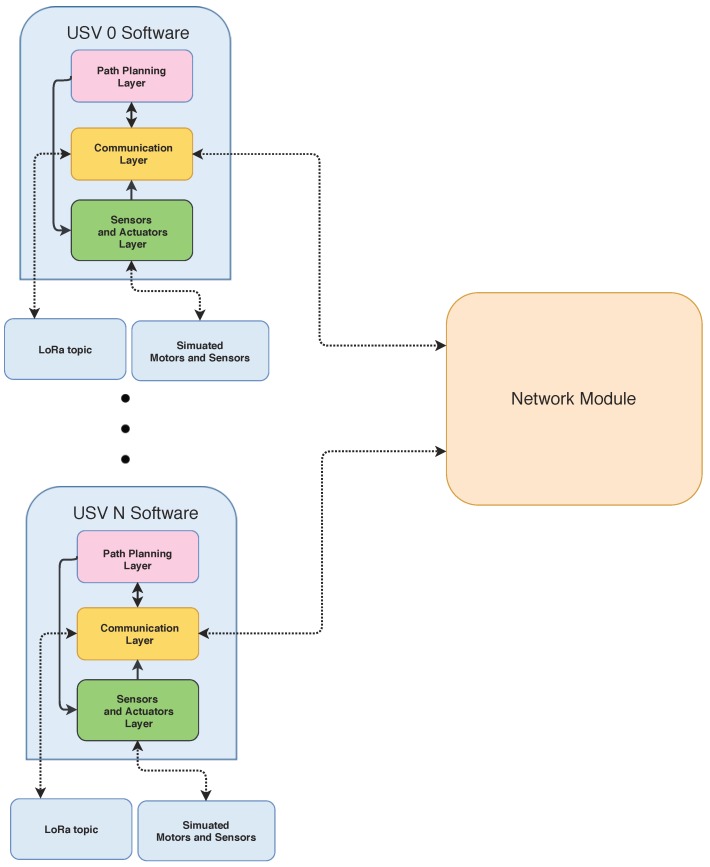
USVs software architecture.

Based on ROS and Gazebo, a realistic simulation environment was developed to facilitate the evaluation of new routing and navigation strategies in controlled aquatic scenarios. This application is able to simulate a realistic aquatic environment with respect to the aquatic sensors on each USV and also the kinodynamics of the motion planning. The simulator uses the same modules of an USV in a real environment, and simulates all the hardware as represented in Figure 7. With respect to the network layer, an additional Network Module was created to have a behavior as the one observed in real wireless networks when applied to aquatic environments.

**Figure 8:**
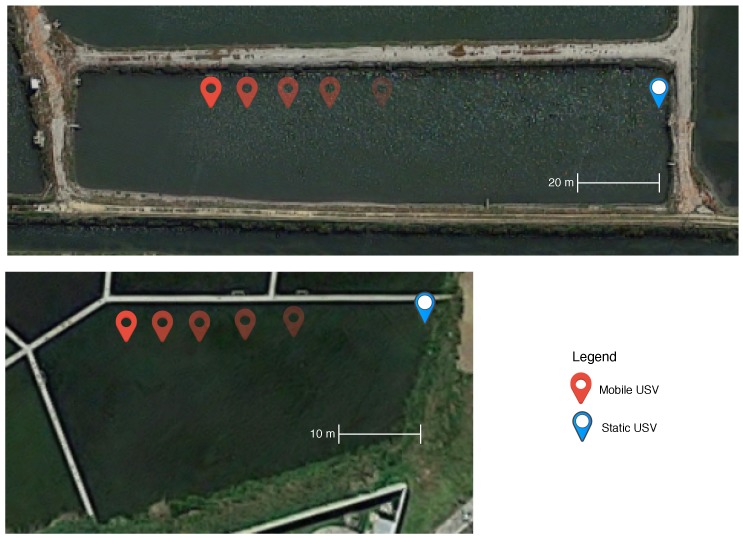
Two aquatic scenarios: (**top**) AlgaPlus scenario; and (**bottom**) the Water Biological Treatment Plant (WBTP) of the University of Aveiro scenario.

To characterize the link quality of an aquatic wireless network to be considered in the Network Module, values of SSI and Achievable Throughput (At) are obtained through experiments in real aquatic scenarios for several aquatic environments. Two distinct scenarios are considered: the AlgaPlus, characterized by tanks influenced by tides and wind, which led to turbulent waters, and where aquaculture is carried out; and the Water Biological Treatment Plant (WBTP) of the University of Aveiro, characterized by standing water, sheltered wind tanks, and has a lot more suspend particles than the other, which leads to reduced visibility. To obtain the aforementioned network metrics, two USVs are used, one mobile and one static. Both water tanks’ USV positions are illustrated in Figure 8.

Measurements were taken in intervals of 5 m for each scenario until we reached 100% of packet loss. For each location, 100 samples were registered. The SSI measurements were obtained using the *iwlist* command while the Achievable Throughput was obtained through WBest technology. Besides the two aquatic scenarios, two antenna positions were also evaluated: with the antenna at the drone level (p0) and 15 cm above the drone level (p15); the drone level is 2 cm above water. For the SSI samples obtained, 5% were filtered out to remove outliers. For both metrics, the shaded area represents the 95% confidence interval.

**Figure 9:**
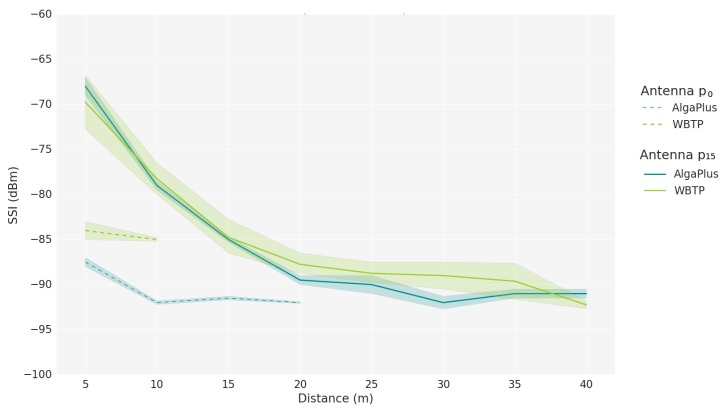
Signal Strength Indicator versus distance.

Figure 9 illustrates the impact of the distance in the SSI values. As expected, this metric is inversely proportional to the distance, i.e., the SSI decreases with the increase of the distance. In addition, for higher distances, the variation of SSI decreases.

The antenna position also affects the signal. When the antenna is at the drone level, the aquatic scenario alters the values of SSI for the same distances. The type of curve is maintained but the values are in different ranges. When the antenna is higher, 15 cm above the drone level, the aquatic parameters no longer influence the SSI values, making it a more preferable location to position the antenna in this type of moving elements.

For the Network Module in the simulator, we modeled the experienced SSI using the results obtained when the antenna is in the p15 position, resulting in a 4th degree equation with a Mean Absolute Error (*MAE*) of 0.217, represented in Equation (6), and valid for distances between 5 and 40 m (expressed by *x*). With this function, we can accurately simulate the SSI value in simulated scenarios. Other fits were tried, such as cubic fit with a *MAE* of 0.269, a quadratic with 1.258, a linear with 2.422, and a logarithmic with 1.179, all of them with higher error than the quartic fit.

(6) $$\begin{array}{c} SSI\_est\left(x\right)=-0.00002\;x^{4}-0.00051\;x^{3}+0.05896\;x^{2}-2.72277\;x-57.5612,\;\;\;\;x\in \left[5,40\right] \end{array}.$$

**Figure 10:**
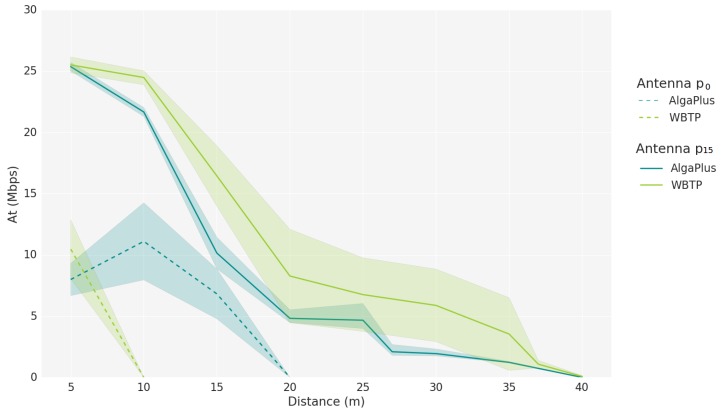
Achievable Throughput versus distance.

Figure 10 represents how the Achievable Throughput is influenced by the distance between the two USVs. As expected, the Achievable Throughput is highly affected by the position of the antennas, especially when compared to the impact of the different aquatic scenarios. When the antenna is at the drone level, the Achievable Throughput is lower. The Bandwidth decreases with the distance, but has a better sensitivity to higher distances, as opposed to the SSI.

The Achievable Throughput obtained through experimentation was used to estimate a delay for each packet in the simulator. The delay was computed by dividing the number of bytes to send by the Achievable Throughput at a certain distance. Once again, using the measurements obtained with the antenna in the p15 position, we can model the Achievable Throughput for this environment using a 4th degree function with a *MAE* of 0.738, given by Equation (7). Other fits were tried, such as cubic fit with a *MAE* of 1.602, a quadratic with 1.602, and a logarithmic with 1.964. The delay also plays a significant role in the link quality estimators, because it is used to determine the bit rate of the connection to be assumed in simulation environments.

(7) $$\begin{array}{c} AT\left(x\right)=-0.00017\;x^{4}-0.01603\;x^{3}+0.472862\;x^{2}-4.20788\;x-14.6655,\;\;\;\;x\in \left[5,40\right] \end{array}.$$

Estimating the Achievable Throughput with a single peer to peer connection may not represent the AT when the channel is being shared by more than one transmitter. Knowing this, in simulation environments, we have to adapt the AT depending on the number of USVs transmitting at the same time and on their distances, which may result in smaller throughputs, and, consequently, higher delays.

## 6. Performance Evaluation

In this section, we describe the simulator and evaluate the performance of the proposed forwarding strategies based on link quality estimations both in simulation and real environments. Section 6.1 describes the simulator, while Section 6.2 describes the scenarios and the tests performed. In Section 6.3, we validate the accuracy of the Link Quality Estimator by comparing its impact in a selected forwarding strategy for both real and simulated scenarios. Then, in Section 6.4, the different proposed forwarding strategies are evaluated and compared in terms of network performance in both real and simulated scenarios. Section 6.5 compares the simulated scenarios with movement.

### 6.1. Simulator Description

**Figure 11:**
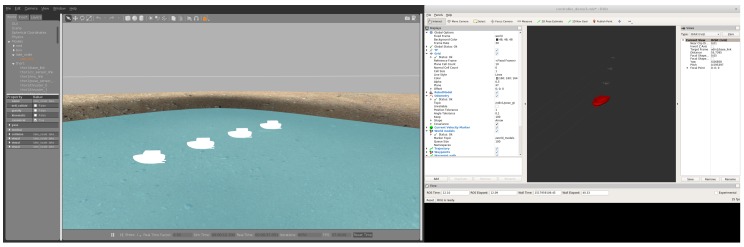
Gazebo on the left and Rviz Editor with an Rviz configuration file to the namespace odin1 on the right.

As mentioned in Section 5, a realistic aquatic simulation environment based on ROS and Gazebo was developed. In Gazebo, a world describes a collection of robots and objects (such as buildings, tables, and lights), and global parameters including the sky, ambient light, and physics properties. After launching all services, nodes, and topics, the simulation starts in Gazebo and Rviz as shown in Figure 11, where the lake world is shown on the left in Gazebo.

For the navigation module, a grid map type is selected. The simulated USV has two thrusters, each with a unique frame, which in turn enables the lookup of the transformation matrix of each thruster and the USV’s body through the use of tf (a package that lets the user keep track of multiple coordinate frames over time). We use a function from Manhães et al. [31] to automatically generate the thruster allocation matrix and translate controls into each thruster’s commands.

To generate the waypoints in the navigation module, a grid map is being updated by the sensors, given the path to a destination point. The position of each robot in the grid map can be updated by the ground truth value given by Gazebo, or the simulated GPS value. The latter can have an associated noise with it.

In Gazebo, a lower real time factor (RTF) does not invalidate the simulation, but a RTF of 1 is desirable, so simulated results can be compared with the results from a real environment with the same modules; however, depending on the machine resources, the RTF can be lower. Step size and update rate are the parameters that can change the RTF. For a computer with an Intel Core i7-7500U, a NVIDIA GEFORCE 940Mx Graphic Card and 8 GB of RAM, Table 4 shows how these parameters affect the RTF.

Another factor that influences the RTF is the graphic display, which can be disabled, allowing a higher number of USVs in the simulation. For the computer described above, this allows simulations with up to 11 USVs. We can also use ROS bag tools for simulating more USVs.

### 6.2. Scenarios Description

To compare the performance of the link quality based forwarding strategies, a set of scenarios are proposed. Two evaluation scenarios (A and B) are conducted in both environments, real and simulated one, while Scenario C, a mobile scenario to prove the adaptability of the routing strategy in dynamic scenarios, is only evaluated in the simulation environment.

**Figure 12:**
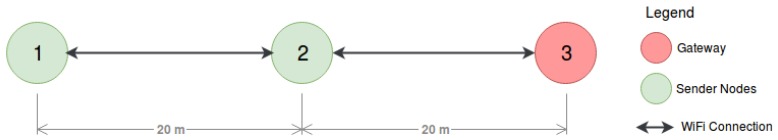
Aquatic Scenario A.

Scenario A is illustrated in Figure 12. This scenario is composed of 3 USVs, where one acts as a Gateway and the other two act as sending nodes. All USVs are fixed and USVs 1 and 2 send packets to USV 3 (the Gateway node). USVs 1 and 3 are not directly connected, which means that USV 2 acts as a connection point between USV 1 and 3. USV 2 is positioned 20 meters away from USVs 1 and 3.

**Figure 13:**
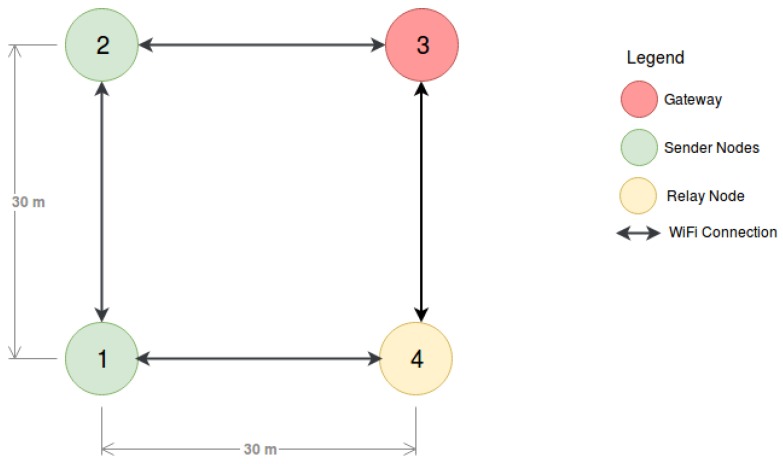
Aquatic Scenario B.

The Scenario B topology is illustrated in Figure 13. In this scenario all USVs are static, similarly to the previous scenario, and USVs 1 and 2 send packets to USV 3 that is acting as Gateway. USVs 3 and 4 do not send packets, which means that USV 4 acts only as a relay node. USVs 3 and 1, and USVs 4 and 2 are not directly connected. In this scenario, the nodes are positioned on the vertices of a 30 m square. The packets transmitted from USV 1 have two possible paths to reach the gateway: Path P1, 1→2→3, and Path P2, 1→4→3.

**Figure 14:**
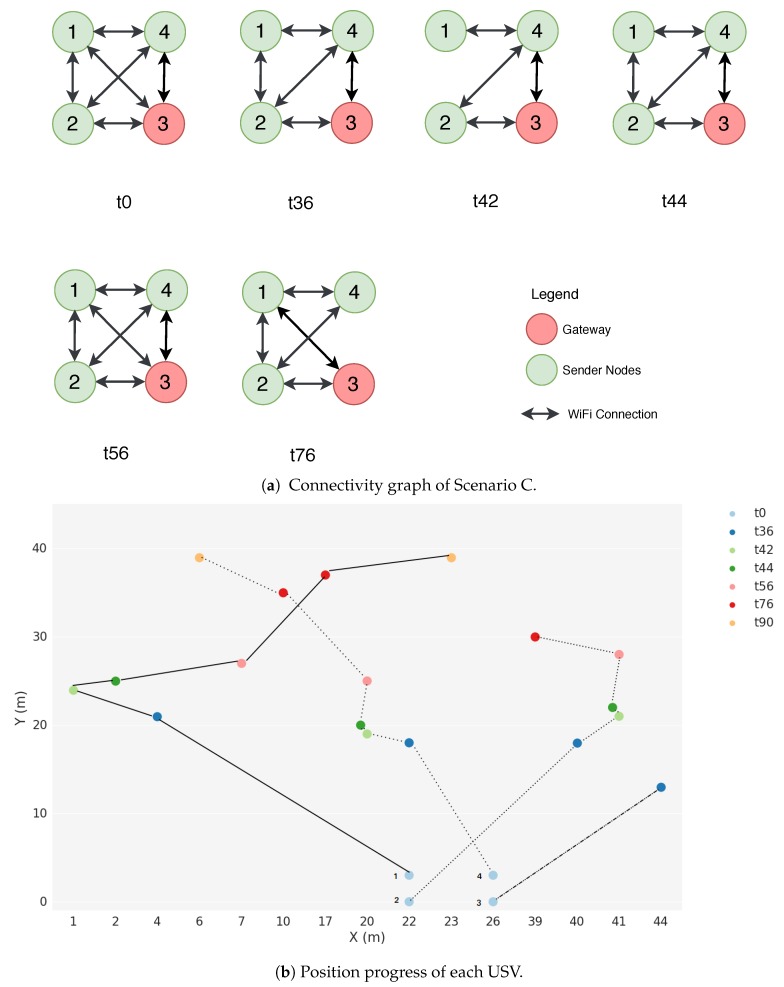
Aquatic Scenario C.

The Scenario C topology is illustrated in Figure 14. In this scenario, all USVs are mobile. USV 3 acts as a Gateway while USVs 1, 2 and 4 generate packets to be transmitted to USV 3. Figure 14a represents the connectivity graph on a set of timestamps observed during the simulation where we can see the available paths for the packets to be transmitted. Figure 14b shows the mobility progress of each USV for the same timestamps whose connectivity map is represented in Figure 14a.

**Figure 15:**
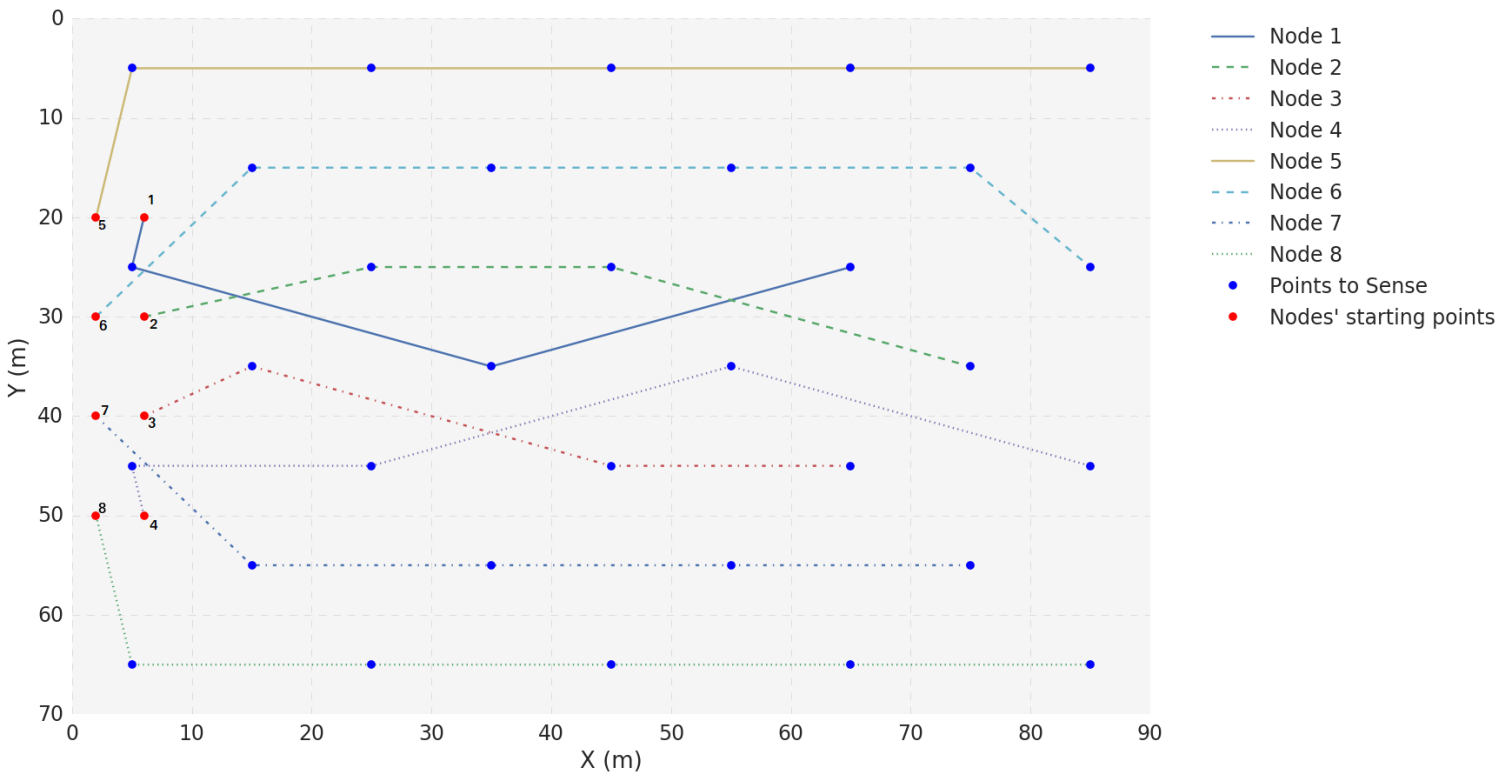
Position progress of each USV in Scenario D.

The Scenario D topology is illustrated in Figure 15. In this scenario, all USVs are mobile. USV 3 acts as a Gateway while USVs 1, 2, 4, 5, 6, 7 and 8 generate packets to be transmitted to USV 3.

**Figure 16:**
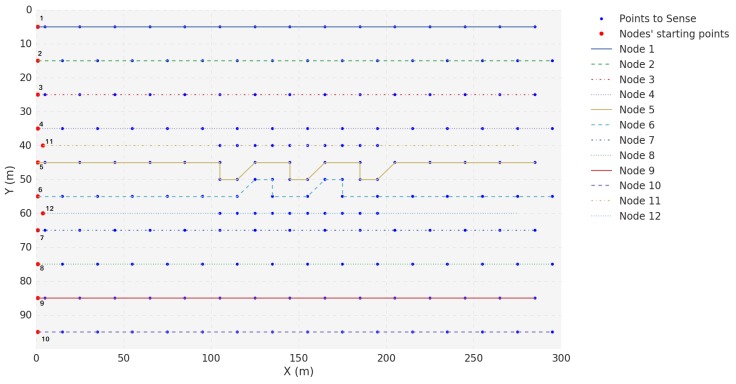
Position progress of each USV in Scenario E.

Finally, the Scenario E topology is illustrated in Figure 16. In this scenario, all USVs are mobile. USV 3 acts as a Gateway while USVs 1, 2, 4, 5, 6, 7, 8, 9, 10, 11 and 12 generate packets to be transmitted to USV 3.

Each topology scenario is used to assess the performance of the proposed forwarding strategies. Table 5 summarizes the characteristics and objectives of each test when in realistic or simulated environment.

### 6.3. Link Quality Estimation Comparison

There is no real link quality metric of reference which other link quality estimators can be compared to. Therefore, to evaluate the metrics utilized in the link estimation, we compare PAmuLQE with a link quality estimation only based on the SSI.

**Figure 17:**
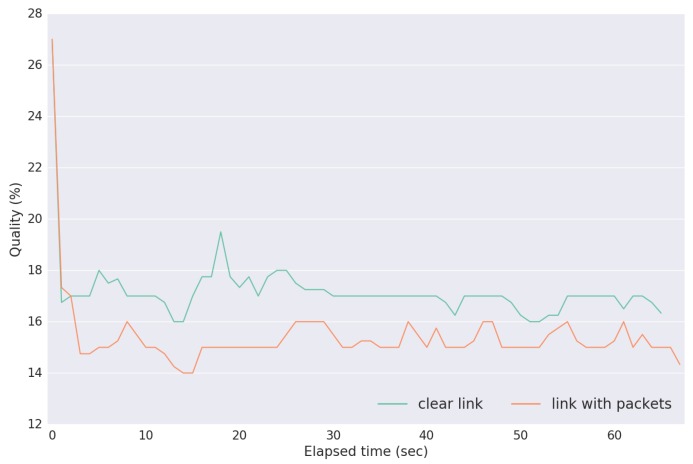
Quality values given by Equation (2), for an unused link (node 4 to node 3) and a link with packets (node 2 to node 3) in Scenario B, test B1.

To measure the quality values, given by Equation (2), we performed test B1, where just USV 2 was sending packets. The evaluation is shown in Figure 17. As expected, PAmuLQE measures a higher quality value on the link with no data packets (node 4 to node 3), due to this estimator taking into account the time that a packet takes from the moment that it is sent to the moment that it is received. Because link 2–3 is being used for transmission besides the announcement packets, this link will introduce a higher delay on each packet, therefore lowering the link quality. This makes path P2 the best and the selected path for delivering packets from node 1 to node 3.

**Figure 18:**
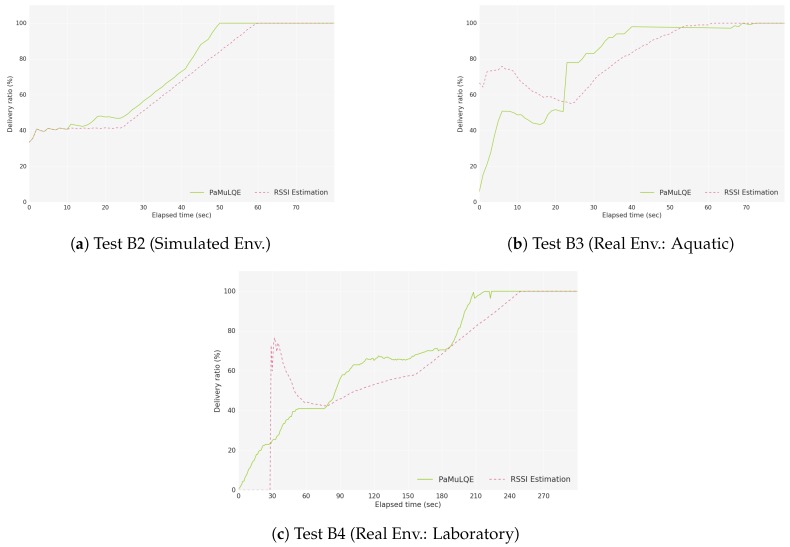
Delivery Ratio for Scenario B: ratio between the delivered data packets to a gateway and the overall data packets.

For the evaluation of the link quality estimator, the delivery ratio is presented in Figure 18 for both tests B2 and B3. In this case, the topology scenario is the same for both scenarios, and USVs 1 and 2 generate packets. The PAmuLQE forwarding strategy is the one selected to assess the impact of the link quality estimator. As expected, when the packets from USV 1 to USV 3 go through path P1, which happens when the LQE is based on RSSI estimation because both paths have the same quality values (because the distance is the same), it takes longer to deliver all packets. However, when PAmuLQE is used, the best path in this case is path P2, which has a higher delivery ratio because this path uses a relay node that does not generate any packet, and therefore more bandwidth to the Gateway is available. Both real and simulated environments present similar behaviors and the same average results.

**Figure 19:**
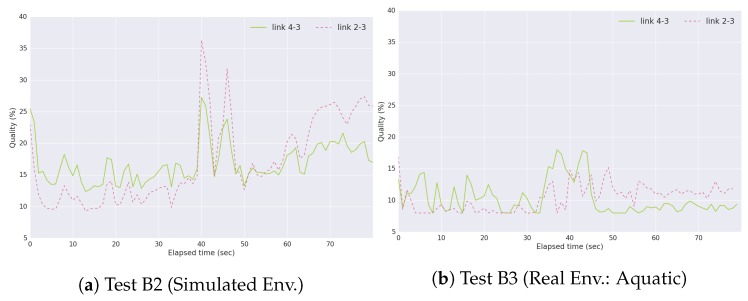
Quality values given by Equation (2) for link 4–3 and link 2–3 in Scenario B.

To evaluate the adaptability of PAmuLQE, we performed tests B2 and B3 and analyzed the quality values measured by Equation (2), which is represented in Figure 19. In the beginning of test B2 and B3, USV 2 forwards packets directly to USV 3, and USV 1 calculates the best path to forward its packets. At this point, the quality values follow the behavior shown in Figure 17, making P2 the chosen path. In the first half of these tests (0–40 s), USV 2 delivers all 100 packets to USV 3, and USV 1 delivers all 100 packets to USV 4. Here, the quality value of link 4–3 is higher, as this connection is not delivering a large amount of packets. When node 2 stops delivering packets, the link 4–3 starts exhibiting a quality value lower than the one of link 2–3, since it stops being used to deliver data packets.

**Figure 20:**
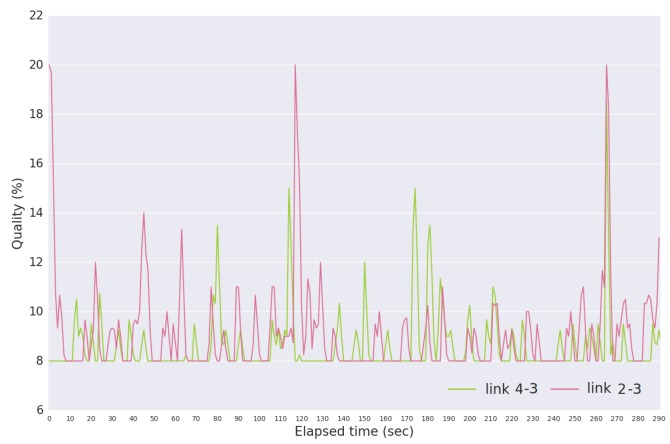
Quality values given by Equation (2) for link 4–3 and link 2–3 in Scenario B, test B4.

In test B3, we increase the amount of packets that USV 2 has to deliver to USV 3, causing links 2–3 and 4–3 to be delivering packets simultaneously. Due to this situation, and as shown in Figure 20, the quality values are closer to each other. Link 4–3 has a mean quality value of 9.1505 and link 2–3 of 8.6520 (difference of 5%).

### 6.4. Link Quality-Based Forwarding Strategies

For the evaluation of the forwarding strategies described in Section 4, the delivery ratio, the mean end-to-end (E2E) delay, and the total network overhead are presented. The proposed strategies are also compared with the well-known Epidemic strategy where each node broadcasts the packet to maximize the delivery ratio and minimize the end-to-end delay, making this a flooding-based protocol. As a result, constraints such as nodes’ buffer size limitation restrict the performance of this strategy. All strategies are evaluated considering the DTN-based architecture mOVE [23].

**Figure 21:**
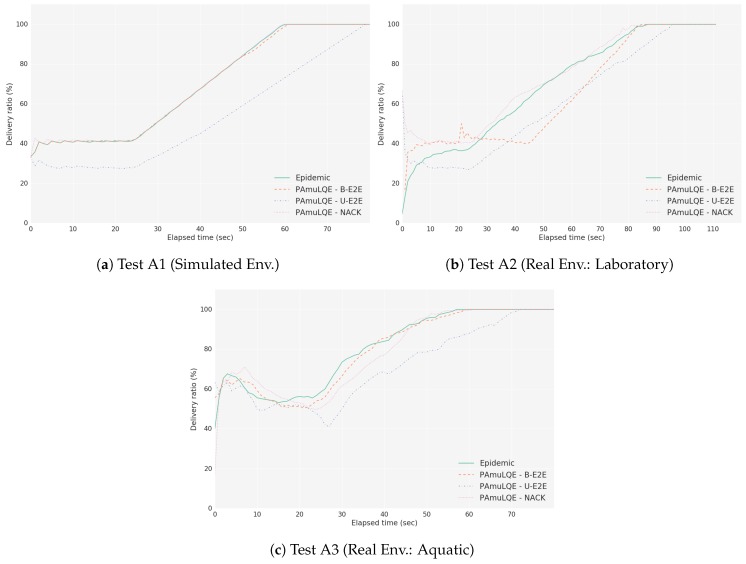
Delivery Ratio for Scenario A. The plots represent the ratio between the delivered data packets to a gateway and the overall data packets.

Figure 21 shows the delivery ratio obtained by each forwarding strategy in Scenario A, for simulated (A1), laboratory (A2) and real environment (A3). As expected, the Epidemic protocol has the highest delivery ratio, because data packets are broadcasted to all nodes in the network without any control. By comparing the three link quality-based strategies with the Epidemic, we can see that PAmuLQE-B-E2E and PAmuLQE-NACK achieve the same delivery ratio, which is also the same as the one achieved by the Epidemic.

**Figure 22:**
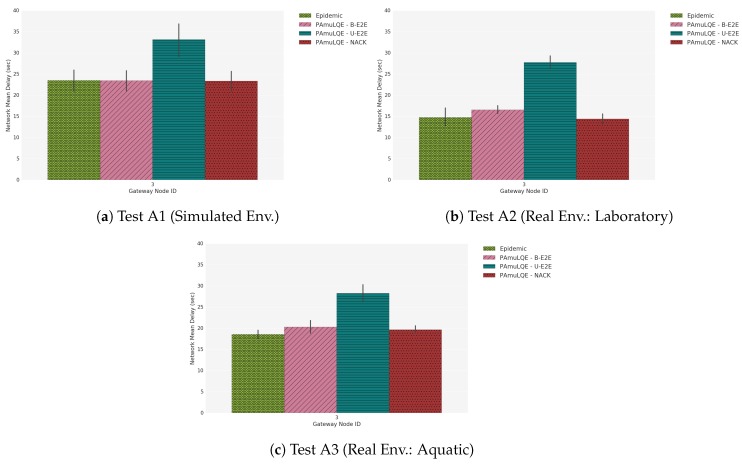
Network Mean Delay for Scenario A. The bar plots represent the amount of time that a data packet takes since its generation until it reaches a gateway.

In agreement with Figure 21, strategies with lower delivery ratios are the ones with higher delivery delay. We observe in Figure 22, where the network E2E delay is illustrated, that PAmuLQE-U-E2E has a higher delay. This is due to acknowledgement messages being sent as data packets, thus having a larger amount of data to send in the storage, delaying the actual data packets.

**Figure 23:**
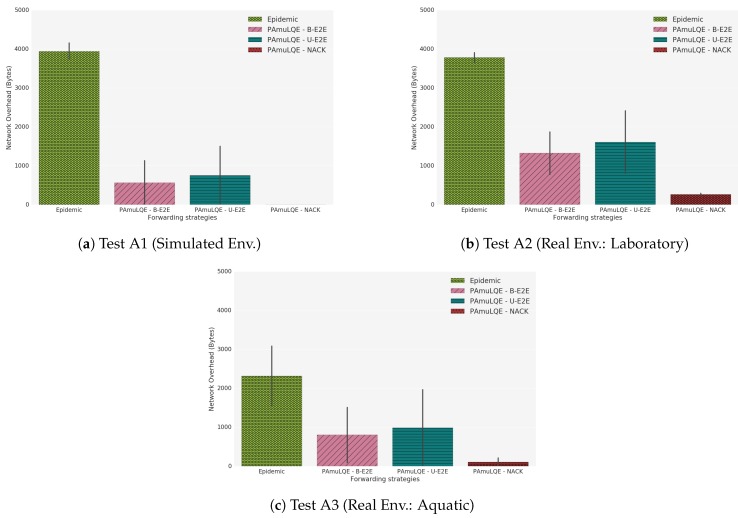
Network Overhead for Scenario A. The bar plots represent the amount of redundant data packets that each strategy introduces in the network.

Regarding the network overhead, Figure 23 shows, as expected, that all proposed variants have lower network overhead when compared to Epidemic, since they do not transmit data packets in broadcast mode. In other words, the proposed variants have a lower replication rate.

Comparing the three tests (A1, A2 and A3), we can observe that the results obtained with the simulator follow the results obtained through real environments. However, we can also observe that the simulated results have smaller variance (and confidence intervals) than the real results, due to the absence of random factors experienced during the real experiments, such as environmental conditions.

### 6.5. Comparison between Mobile and Static Scenarios

**Figure 24:**
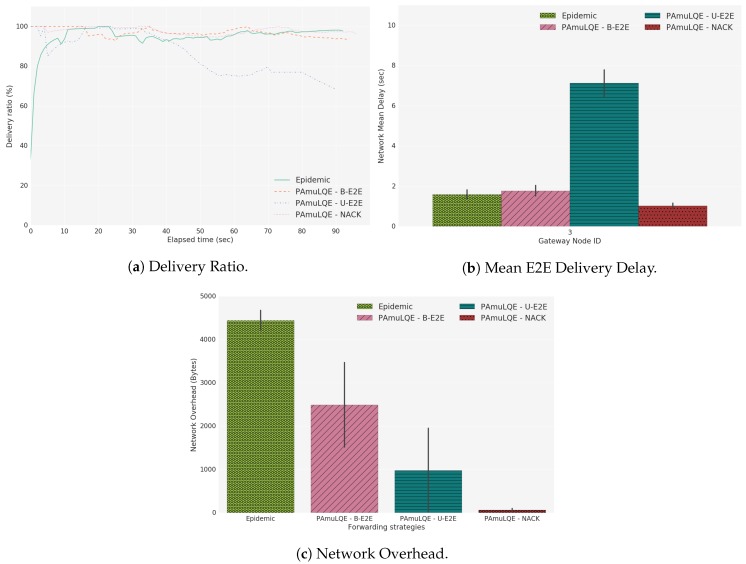
Test C1, Scenario C.

For the evaluation of the proposed link quality-based forwarding strategies in a mobile scenario, the delivery ratio, the mean E2E delivery delay, and the total network overhead experienced in test C1 are presented. As shown in Figure 24a, the delivery ratio has similar behavior compared to Test A1 and Test A2, i.e., PAmuLQE-U-E2E has a lower delivery ratio, and the other proposed variants have a similar ratio compared to Epidemic. In accordance, Figure 24b shows a higher delay for PAmuLQE-U-E2E.

For scenarios with a higher number of USVs, PAmuLQE-B-E2E starts showing a higher network overhead due to the broadcast of the ACK, and because a higher number of nodes can re-send the same packet (Figure 24c). The only disadvantage of PAmuLQE-NACK in mobile scenarios is that, when a USV with a packet navigates away from the swarm, and the only available connection is to a USV where the packet already passed, this packet is lost. This happens because this USV is sending the packet to a USV that already had it, which makes it unable to reach the gateway until a new connection is established with a USV that has never received the same packet.

**Figure 25:**
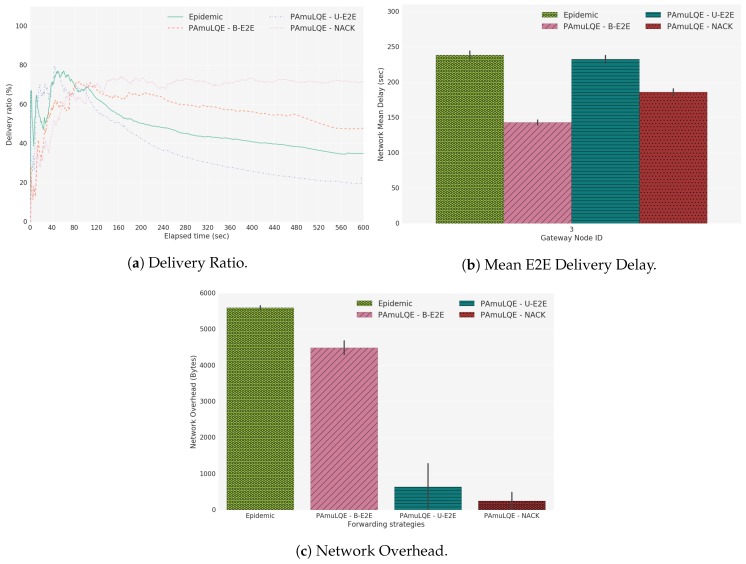
Test D1, Scenario D.

**Figure 26:**
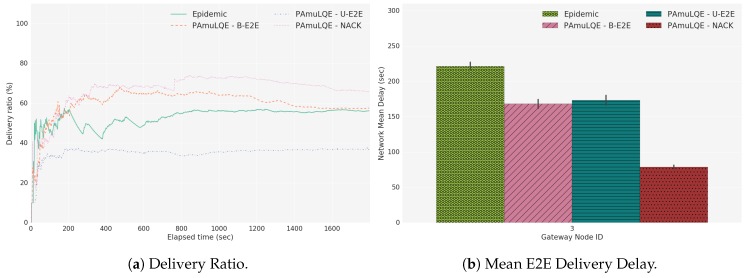

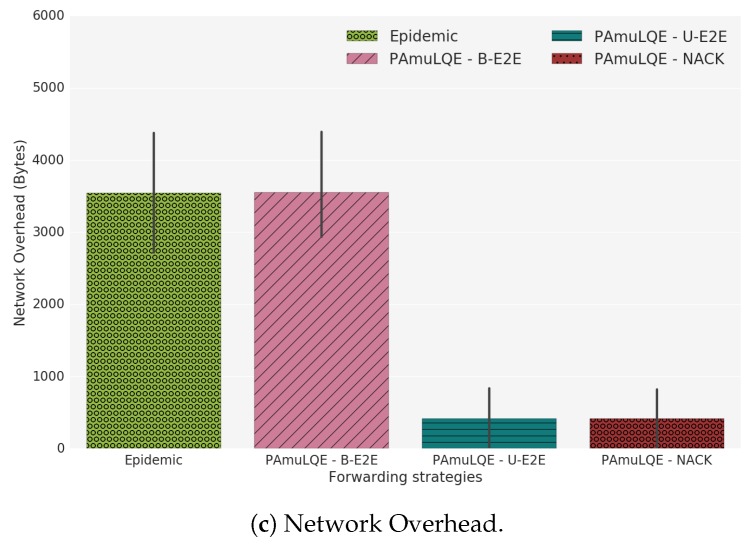
Test E1, Scenario E.

Two more experiments were conducted, D1 and E1, to compare the proposed strategies in scenarios with more USVs and more time of simulation. The results are shown in Figure 25 and Figure 26.

As can be observed in the obtained results, when increasing the number of nodes, the epidemic strategy has no longer a high delivery ratio, a fact that occurs because in this strategy each packet is blindly replicated by its neighbors, therefore increasing the number of copies in the network, and, consequently, the number of duplicated packets received by the gateway.

The PAmuLQE-B-E2E also starts lowering its delivery ratio when the number of network nodes increases because the number of acknowledgements that are sent in broadcast mode also increases, therefore reducing the available bandwidth for data transmissions. Moreover, because this strategy has a mechanism of retransmitting a packet if an acknowledgement is missing, when the number of nodes increases, the number of unnecessary packet retransmissions also increases. For this reason, overall, the best strategy in large scenarios in the PAmuLQE-NACK, since the neighbor acknowledgements provide the existence of one packet in the network at each time while achieving a high delivery ratio.

## 7. Conclusions

In this work, a low-cost aquatic mobile sensing platform based on USV swarms was proposed. A simulation environment was proposed based on ROS and Gazebo where one could test proposed routing strategies. A network module was developed where the results of the experiments in real events have the same behavior as in the simulated environment. Regarding the proposed routing strategies, it has been verified that an LQE based on RSSI estimation and Bit Rate has better delivery ratio than that based only on RSSI estimation. It can also be concluded that, from the various proposals, which vary in the way the packet is acknowledged, none is better for all the scenarios. In a static scenario, PAmuLQE-NACK is indeed the best. This is not the case when dealing with mobile scenarios, as strategy can lead to the loss of the packet by expiration. For the mobile scenarios with a small number of USVs, PAmuLQE-B-E2E is the best strategy, because it combines a higher delivery ratio with a lower overhead. For larger numbers of USVs, PAmuLQE-NACK is more suitable since it has a lower overhead and higher delivery ratio.

As future steps, we aim to further assess our platform with different real mobile scenarios, over larger evaluation periods and considering a more dense and populated network, providing the organization and cooperation of several swarm clusters. Another point to be tackled in the near future is the evaluation of the Cooperative Path Planning and Trajectory Modification modules when link failures occur.

## Figures and Tables

**Table 1:** Forwarding Strategies overview.

Name	Type	Single/Multiple Copy	Replication Rate	Passive/Active Monitoring
Direct contact	Direct	S	N/A	N/A
Epidemic	Flooding	M	Very high	N/A
Drone-Quality Delay Tolerant Routing Approach	Probabilistic	S/M	Medium	Hybrid
Q-PRoPHET	Probabilistic	S/M	Medium	Passive with Overhearing
QoN-BSW	Probabilistic	M	High	Active
HPR	Probabilistic	N/A	N/A	Hybrid
PAmuLQE	Probabilistic	S/M	Low/Medium	Passive

**Table 2:** Raspberry Pi 3 Model B specifications.

**Processor**	1.2 GHz 64-bit quad-core ARMv8 CPU
**Memory RAM**	1 GB
**Wi-Fi Networking**	2.4 GHz 802.11n Wireless LAN

**Table 3:** Sensors description.

Name	Measured Parameters	Interface
GPS-MTK3339 [35]	Latitude and Longitude	UART
IMU SEN0140 [36]	Velocity, Orientation, Gravitational forces, and Pressure	I2C
Conductivity Kit K1.0 [37]	Electrical Conductivity	I2C
pH-SEN0161 [38]	pH level	Analog
Turbidity-SEN0189 [39]	Levels of turbidity (light is used to detect suspended particles in water by measuring the light transmittance and scattering rate)	Analog
Ultrasonic-SEN0208 [40]	Distance (both depth and obstacles)	Digital
Depth/Pressure-BAR30 [41]	Depth and pressure	I2C
Liquid Level-SEN0205 [42]	Whether the probe is submerged or not	Digital
Temperature-DS18B20 [43]	Water temperature	1-wire

**Table 4:** Real time factor depending on number of USVs, step size and update rate.

Number of USVs	Step Size	Update Rate	RTF
4	0.001	1000	0.7
4	0.01	100	8
7	0.01	100	1

**Table 5:** Tests description.

Label	Environment (Simulation, Laboratory or Aquatic)	# Packets Sent	Objective	Notes
A1	S	Node 1: 100 Node 2: 100	Compare the several strategies in a simulation environment.	
A2	L	Node 1: 100 Node 2: 100	Compare the several strategies in a laboratory environment.	
A3	A	Node 1: 100 Node 2: 100	Compare the several strategies in an aquatic environment.	This experiment was developed in the WBTP.
B1	L	Node 1: 0 Node 2: 100	Test the quality measurements on the links connected to the gateway.	
B2	S	Node 1: 100 Node 2: 100	Test the performance of PAmuLQE compared to a LQE based on RSSI. Test the adaptability of PAmuLQE to evaluate the network.	LQE based on RSSI Estimation uses path P1 (Worst case scenario) when delivering packets from node 1 to node 3, because both paths have the same quality values. The algorithm chooses the first path computed in case of a tie.
B3	A	Node 1: 100 Node 2: 100	Test the performance of PAmuLQE compared to a LQE based on RSSI. Test the adaptability of PAmuLQE to evaluate the network.	LQE based on RSSI Estimation uses path P1 (Worst case scenario) when delivering packets from node 1 to node 3, because both paths have the same quality values. The algorithm chooses the first path computed in the case of a tie.
B4	L	Node 1: 200 Node 2: 500	Test the performance and resembles in real scenarios with simulated ones.	LQE based on RSSI Estimation uses path P1 (Worst case scenario).
C1	S	All nodes except 3 (gateway) generate 2 packets every second, during 1.5 min.	Test the several strategies in a mobile scenario.	
D1	S	All nodes except 3 (gateway) generate 1 packet every second, during 10 min.	Test the several strategies in a mobile scenario with a larger USV swarm.	
E1	S	All nodes except 3 (gateway) generate 1 packet every 3 s, during 30 min.	Test the several strategies in a mobile scenario with a larger USV swarm.	
